# Meniscal and Chondral Injury Patterns in Athletes With Anterior Cruciate Ligament Tear

**DOI:** 10.7759/cureus.49282

**Published:** 2023-11-23

**Authors:** Sushmita Kushwaha, Firoz A Khan, Chethan R, Pramod Kumar, Shorya Singh

**Affiliations:** 1 Department of Sports Medicine, Pandit Bhagwat Dayal Sharma Post Graduate Institute of Medical Sciences, Rohtak, IND; 2 Department of Sports Medicine, Sports Injury Centre, Safdarjung Hospital, New Delhi, IND; 3 Department of Sports Medicine, Inspire Institute of Sport, Mysore, IND

**Keywords:** injury pattern, athletes, knee arthroscopy, chondral lesions, meniscal tear, anterior cruciate ligament

## Abstract

Anterior cruciate ligament (ACL) tears are a prevalent and debilitating injury among athletes, often accompanied by concurrent meniscal and chondral injuries. This study aimed to present a comprehensive investigation into the patterns and prevalence of meniscal and chondral injuries in athletes with ACL tears. This is a cross-sectional study conducted on 600 athletic patients with ACL tears planned for reconstruction in a duration of five years. A combination of advanced imaging techniques, arthroscopic evaluations, and clinical data was used to provide a comprehensive understanding of the injury profiles of the participant athletes. Those findings were duly recorded and analyzed accordingly.

Out of 600 patients, 67% (402) had at least one meniscal or chondral injury while the rest 33% (198) had isolated ACL injuries only. Of the patients, 18% (108) were those who had both meniscal and chondral injuries present. Amongst the 57% (342) of patients who had meniscal injuries, injuries to the medial meniscus, lateral meniscus, and both the meniscus were present in 51% (175), 32% (109), and 17% (58) of patients, respectively. Amongst all associated meniscal injuries (n_1_ = 404), around 52% (210) tears were present in the body of the meniscus, 31% (125) in the posterior horn, and 17% (69) in the anterior horn. Overall, it was noted that 22.77% (92) of meniscal tears were bucket handle tears of the medial meniscus, 16.08% (65) were complex tears of the posterior horn of the lateral meniscus, and 9.60% (39) were complex tears of the posterior horn of the medial meniscus.

Amongst 600 patients, 28% (168) of patients had at least one chondral injury present in association with ACL tear. Further, amongst the total number of chondral lesions reported (n_2_ =297) in ACL-deficient knees, around 55% (163) of lesions were located on medial femoral condyle, 10% (30) were located on undersurface of patella, 10% (30) were global changes, 7% (20) were on lateral femoral condyle, and 5% (15) were located on medial articulating surface of knee. A total of 61% (181) of chondral lesions were grade II, 21% (62) were grade III, 10%(30) were grade IV, and the least noted were 8% (24) grade I chondral lesions.

The study concludes that medial meniscus injury was the most common meniscal injury in ACL-deficient knees and the bucket handle tear of the medial meniscus was the most common type of meniscal tear followed by the complex tear of the posterior horn of the medial meniscus. Further, the study also concludes that the medial femoral condyle is the most common site of chondral lesions in ACL-deficient knees.

## Introduction

Anterior cruciate ligament (ACL) tears are a prevalent and debilitating injury among athletes, particularly those engaged in high-impact sports [1,2]. While the focus has traditionally been on the primary ligamentous damage, emerging evidence suggests a significant association between ACL tears and concomitant meniscal and chondral injuries [3-9]. Understanding the intricate interplay of these structures is crucial for devising effective management strategies and optimizing long-term outcomes for athletes.

The menisci and articular cartilage play pivotal roles in knee joint stability, load distribution, and shock absorption [10-12]. In the context of ACL tears, the dynamics of these structures become even more critical. Yet, there is a gap in the current literature regarding the specific patterns and prevalence of meniscal and chondral injuries in athletes with ACL tears. The multifaceted nature of these injuries presents a challenge for clinicians, as the intricacies of their interdependence are not yet fully elucidated. This knowledge deficit hampers our ability to tailor treatment approaches, potentially leading to suboptimal recovery and increased risk of long-term complications.

This research aims to bridge existing gaps in our knowledge by conducting a comprehensive analysis of meniscal and chondral injury patterns in athletes with ACL tears by examining a diverse cohort of athletes across various sports. Understanding the nuanced relationships between ACL tears, meniscal injuries, and chondral damage is pivotal for tailoring effective treatment strategies that address the holistic impact of these injuries on an athlete's joint health and overall athletic career.

## Materials and methods

This study was a cross-sectional study conducted on 600 athletic patients of ACL tear visiting Sports Injury Centre, Vardhman Mahavir Medical College (VMMC) & Safdarjung Hospital (SJH) from 2017 to 2021. Ethical approval from the Institutional Ethics Committee (IEC) of Vardhman Mahavir Medical College & Safdarjung Hospital was taken prior to the start of the study (IEC/VMMC/SJH/Thesis/October-2015).

All the patients visiting the outpatient department of the hospital with complaints of acute or chronic knee pain, instability, swelling, or locking were examined clinically. Radiological investigations like X-rays and magnetic resonance imaging of the involved knee were done to confirm the diagnosis. Patients above 16 years of age, with complete ACL tears with or without any evidence of meniscal tear or cartilage lesions, were included in the study. Whereas patients with a multi-ligamentous knee injury, ACL graft tear, ACL avulsion injury, and generalized ligamentous hyperlaxity were excluded from the study. Likewise, 600 patients with clinically and radiologically diagnosed ACL tears planned for arthroscopic ACL reconstruction were recruited in this study during the span of five years. Written informed consent was taken from each participant before enrolment into the study.

All the 600 participants underwent arthroscopic ACL reconstruction of the involved knee as planned before enrolment into the study. During their arthroscopic knee surgery, the status of the meniscus and articular cartilage was documented. Meniscal findings like the presence or absence of meniscal tear, side, site, and type of meniscal tear were noted. Accordingly, the presence of chondral lesions was documented with respect to the location and grade of the lesion. The International Cartilage Repair Society (ICRS) cartilage lesion classification system was used to grade the cartilage lesions from grade 0 to grade 4. Grade 0 is given to normal cartilage, grade 1 to the superficial lesions, grade 2 to the lesions extending down to <50% of cartilage depth, grade 3 to the cartilage defect extending down to more than >50% of the cartilage including blisters, and grade 4 were given to the osteochondral injuries, lesions extending just through the subchondral bone plate or deeper defects down into the trabecular bone.

Statistical analysis

Data were entered into Microsoft Office Excel 2019 spreadsheets (Microsoft Corporation, Redmond, WA), and the analysis was conducted utilizing the licensed Statistical Package for Social Sciences (SPSS) version 27.0 (IBM, Armonk, NY). Demographic and arthroscopic findings were subjected to descriptive statistical analysis. The occurrences of meniscal and chondral lesions were computed independently. Numerical and percentage representations were employed for categorical variables, whereas continuous variables were conveyed as mean ± standard deviation and median.

## Results

**Figure 1:**
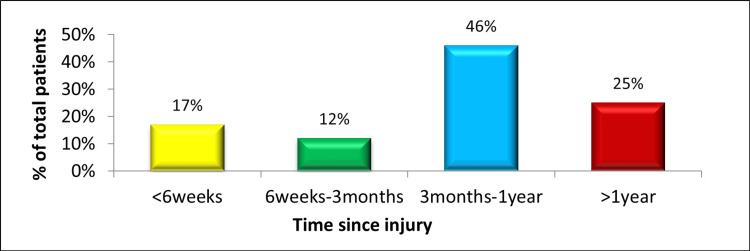
Frequency of patients with respect to time since knee injury. The data have been represented as a percentage (%) of total participants (N = 600).

This cross-sectional study investigated 600 patients with complete ACL tears who were scheduled for ACL reconstruction. The study cohort comprised 540 (90%) males and 60 (10%) females, ranging in age from 16 to 45 years, with a median age of 27 years and an interquartile range of 22-33 years. The frequency of patients with respect to time since knee injury is presented in Figure 1.

**Figure 2:**
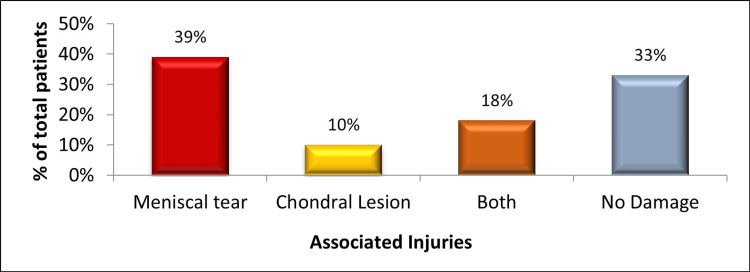
Frequency of associated injuries in ACL-deficient knees. The data have been represented as a percentage (%) of total participants (N = 600). ACL: anterior cruciate ligament.

Within the cohort of 600 participants with ACL tears who underwent arthroscopic ACL reconstruction, the study revealed that approximately 67% (402) of patients exhibited at least one meniscal or chondral injury concomitant with their ACL tear. Among the total participants, 33% (198) had no additional knee injuries aside from the ACL tear. Specifically, 39% (234) of patients displayed only meniscal injuries, while 10% (60) had solely chondral injuries in conjunction with their ACL tear. Additionally, 18% (108) of patients were identified as having both meniscal and chondral injuries, as illustrated in Figure 2.

Meniscal injury pattern in ACL-deficient knee

**Figure 3:**
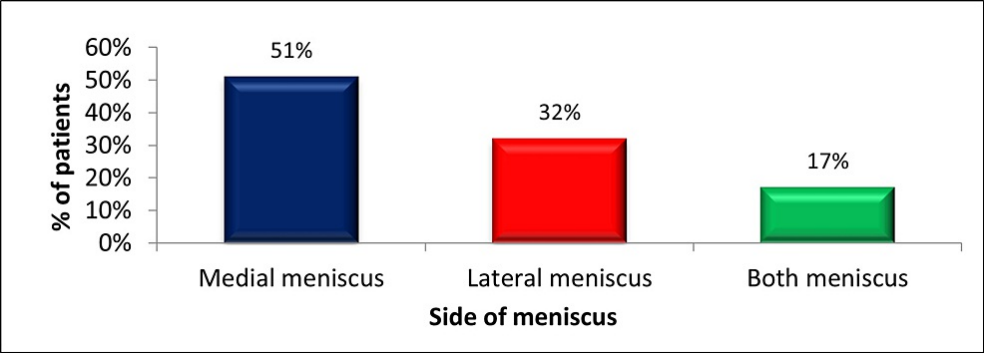
Side of meniscal tear in ACL-deficient knees. The data have been represented as a percentage (%) of participants having associated meniscal tears (n = 342). ACL: anterior cruciate ligament.

Out of 57% (342) of patients having associated meniscal tear in ACL-deficient knee, it was found that around 51% (175) of patients had a medial meniscal tear, around 32% (109) had a lateral meniscal tear, and around 17% (58) of patients had both medial and lateral meniscal tears, as illustrated in Figure 3.

**Figure 4:**
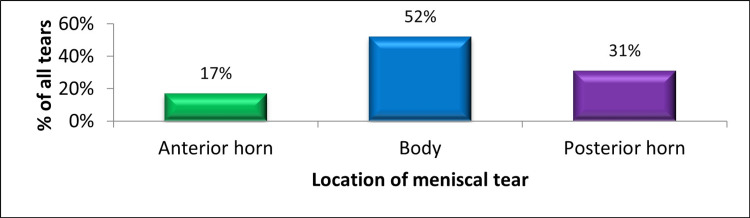
Location of meniscal tear in ACL-deficient knees. The data have been represented as a percentage (%) of all meniscal tears (n1 = 404). ACL: anterior cruciate ligament.

Out of all tears observed within the meniscus (n1 = 404), 52% (210) of tears were located in the body of the meniscus, followed by the posterior horn in around 31% (125) and anterior horn in around 17% (69), as shown in Figure 4.

**Figure 5:**
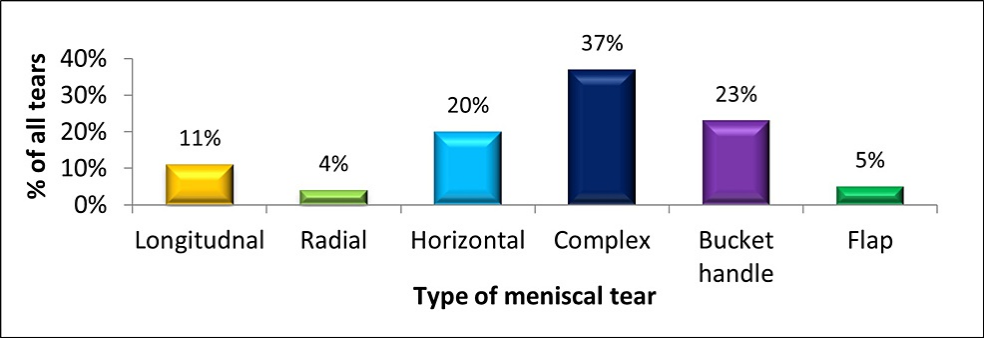
Type of meniscal tears in ACL-deficient knees. The data have been represented as a percentage (%) of all meniscal tears (n1 = 404). ACL: anterior cruciate ligament.

It was also observed that out of all types of meniscal injuries noted (n1 = 404), around 37% (150) of tears were complex type, 23% (93) were bucket handle tears, 20% (81) were horizontal, followed by 11% (44) longitudinal, 5% (20) flap, and around 4% (16) were radial tears, as illustrated in Figure 5.

**Figure 6:**
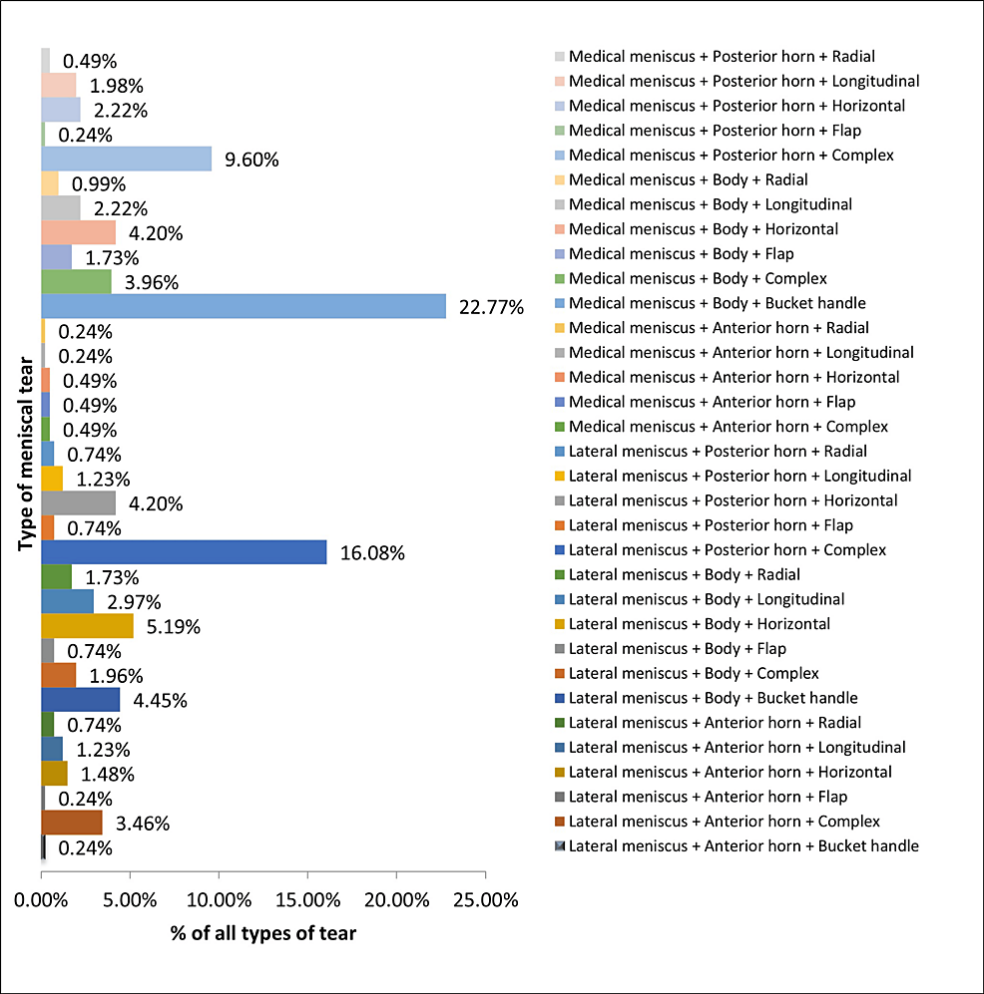
Detailed meniscal injury pattern in ACL-deficient knees. The data have been represented as a percentage (%) of all meniscal tears (n1 = 404). ACL: anterior cruciate ligament.

Looking upon the complete profile of associated meniscal injury (n1 = 404) patterns in athletes with ACL tears, it was found that the most common type of meniscus tear in an ACL-deficient knee in athletes is the bucket handle tear of the body of medial meniscus (22.77%, 92), followed by a complex tear of the posterior horn of the lateral meniscus (16.08%, 65), complex tear of the posterior horn of the medial meniscus (9.60%, 39), horizontal tear of the body of the lateral meniscus (5.19%, 21), bucket handle tear of the body of the lateral meniscus (4.45%, 18), horizontal tear of the body of the medial meniscus (4.20%, 17), horizontal tear of the posterior horn of the lateral meniscus (4.20%, 17), and so on, as illustrated in Figure 6.

Chondral injury pattern in ACL-deficient knee

**Figure 7:**
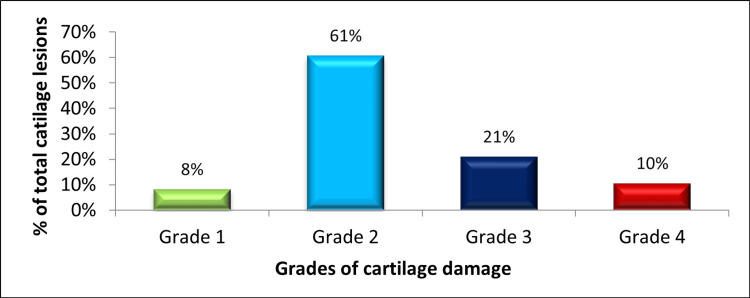
Grades of chondral lesions (as per ICRS) in ACL-deficient knees. The data have been represented as a percentage (%) of total chondral lesions (n2 = 297). ACL: anterior cruciate ligament; ICRS: International Cartilage Repair Society.

Amongst the 600 athletic patients with ACL tears, 168 (28%) patients had the presence of one or more chondral lesions in their ACL-deficient knee. Those lesions were graded on the basis of the International Cartilage Repair Society grading from grade 1 to grade 4. Amongst the total number of cartilage lesions present (n2 = 297), the majority of the lesions, around 61% (181), were grade 1, followed by grade 3 in around 21% (62) and grade 4 in around 10% (30), and the least were grade 1 lesions in around 8% (24), as shown in Figure 7.

**Figure 8:**
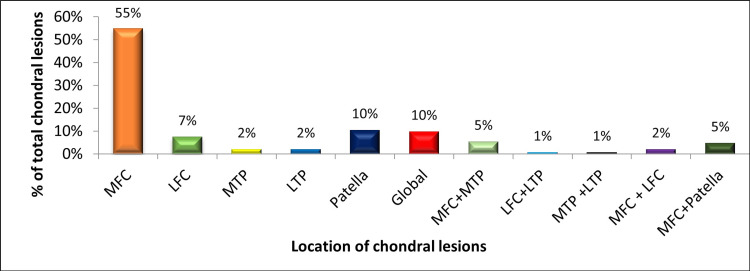
Location of chondral lesions in ACL-deficient knees. The data have been represented as a percentage (%) of total chondral lesions (n2 = 297). ACL: anterior cruciate ligament; MFC: medial femoral condyle; LFC: lateral femoral condyle; MTP: medial tibial plateau; LTP: lateral tibial plateau.

Out of all the associated cartilage lesions (n2 = 297) present in the ACL-deficient knees of 128 patients, around 55% (163) of lesions were located on the medial femoral condyle (MFC), around 10% (30) on the undersurface of the patella, around 10% (30) cases were those of global chondral changes, around 7% (20) on lateral femoral condyle (LFC), 5% (15) on the medial articulating surface of the knee (MFC and medial tibial plateau (MTP)), 5% (15) on MFC and undersurface of the patella together, around 2% (6) lesions on the MTP and lateral tibial plateau (LTP) separately, and around 1% (3) separately on the lateral articulating surface of femur and tibia (LFC and LTP) and articulating surface of tibia (MTP and LTP), as illustrated in Figure 8.

## Discussion

The incidence of ACL tear is increasing day by day due to greater participation in sports and recreational activities by the general population. Its reconstruction is amongst the most commonly performed orthopedic procedures. However, outcomes of ACL surgery are not uniformly excellent due to the presence of additional meniscal and articular cartilage lesions. The nuanced patterns and prevalence of these secondary injuries in athletes with ACL tears remain a subject of limited understanding and ongoing investigation. The present study conducted on 600 athletic patients of ACL included an analysis of injury prevalence and the anatomical location of meniscal and chondral injuries.

In our research, we found that the concurrent occurrence of associated meniscal and chondral injuries in ACL-deficient knees was 67%, closely aligning with the prevalence of additional meniscal and chondral injuries in ACL-deficient knees reported in previous studies [3-5,9,13].

In our study, we observed that 57% of cases involved associated meniscal injuries. Notably, medial meniscal tears were more predominant, constituting 51%, in contrast to lateral meniscus tears, which accounted for 32%. The overall incidence of both medial and lateral meniscus tears was 17%. Upon further examination, it was found that among various types of meniscal tears, the most frequent was the bucket handle tear of the medial meniscus (22.77%), followed by the complex tear of the posterior horn of the lateral meniscus (16.08%) and the complex tear of the posterior horn of the medial meniscus (9.60%). Previous studies have reported a wide range of meniscal injury rates in ACL tear patients, ranging from approximately 22% to 86%. Additionally, these studies have consistently found meniscal tears to be more prevalent than lateral meniscal tears, with bucket handle tears and complex tear types being more common [3-5,9,13-18].

In our study, the overall occurrence rate of articular cartilage lesions was 28%, and the medial femoral condyle (55%) is the predominant location for chondral lesions in the ACL-deficient knee, followed by the undersurface of the patella (10%), global chondral lesions (10%), the lateral femoral condyle (7%), and the medial compartment (5%), aligning closely with rates reported in similar previous studies, which have ranged from 11% to 43% [3-9,19-22]. Additionally, the medial femoral condyle was more common to have chondral lesions in ACL-deficient knee [9]. Further, our study found that the most frequently observed grade of chondral lesions is grade 2 (61%), followed by grade 3 (21%), grade 4 (10%), and grade 1 (8%).

This research endeavors to bridge this knowledge gap by delving into the distinct meniscal and chondral injury patterns prevalent in athletes grappling with ACL tears. Through a comprehensive synthesis of clinical observations, advanced imaging modalities, and outcome assessments, we seek to unravel the complex inter-relationships among these structures. Recognizing the significance of timely and precise interventions, our exploration aims to furnish clinicians, sports medicine practitioners, and rehabilitation specialists with a detailed map of the injury landscape.

Our study has its limitations. We cannot determine whether meniscal and chondral injuries were present prior to ACL injury, occurred at the time of injury, or developed after ACL tear. Additionally, the study exclusively involves a sporting population and lacks data regarding the non-sporting population. Therefore, the results cannot be generalized to the broader population.

## Conclusions

The study establishes that meniscal and chondral injuries are highly prevalent among individuals with ACL tears, providing a detailed profile of these associated injuries. This research article aims to equip orthopedic surgeons, sports medicine practitioners, and researchers with valuable insights for making more informed decisions in the diagnosis and treatment of ACL injuries in athletes. Additionally, it contributes to the ongoing discourse on injury prevention and rehabilitation strategies, with the goal of improving the overall well-being and longevity of athletes. Future research could explore the prevalence of these associated injuries in non-sporting populations and investigate factors predicting these injuries in knees with ACL deficiency.
